# Population structure of blackfin tuna (*Thunnus atlanticus*) in the western Atlantic Ocean inferred from microsatellite loci

**DOI:** 10.1038/s41598-022-13857-z

**Published:** 2022-06-14

**Authors:** Eric A. Saillant, Patricia L. Luque, Emily Short, Luca Antoni, Lionel Reynal, Cedric Pau, Freddy Arocha, Pollyana Roque, Fabio Hazin

**Affiliations:** 1 School of Ocean Science and Engineering, Gulf Coast Research Laboratory, University of Southern Mississippi, Ocean Springs, MS 39564 USA; 2 IFREMER Délégation de Martinique, 97231 Le Robert, La Martinique, France; 3 Instituto Oceanográfico de Venezuela, Universidad de Oriente, Cumana, 6101 Venezuela; 4 UFRPE-Universidade Federal Rural de Pernambuco, Rua Dois Irmãos, s/n, Recife, PE Brazil; 5 Present Address: AZTI, Marine Research, Basque Research and Technology Alliance (BRTA), Pasaia, Gipuzkoa Spain

**Keywords:** Molecular ecology, Marine biology

## Abstract

The blackfin tuna, *Thunnus atlanticus*, is a small tropical tuna exploited by recreational and commercial fisheries in various parts of its range. Information on stock structure is needed to develop management plans for this species but is currently lacking. In this work, 470 blackfin tuna from nine geographic populations were assayed at 13 homologous microsatellite markers to provide a first assessment of stock structure across the species range. The overall divergence among locality samples was very low (overall FST = 0.0004) indicating high connectivity of blackfin tuna across their range. No clear grouping of localities in differentiated units was inferred but structuring followed a weak isolation by distance pattern (r = 0.16, P = 0.032). Pairwise exact tests and spatial analysis of molecular variance suggested divergence of the sample collected offshore Baía Formosa (Brazil) possibly reflecting reproductive isolation of Brazilian populations from those in the Caribbean region and further north. Further study of the status of Brazilian populations and the transition between this region and the Caribbean is warranted. Cryptic subdivision within the Northern Hemisphere part of the range is possible and should be evaluated using increased marker density and a more comprehensive geographic coverage.

## Introduction

Understanding the structure and connectivity of populations is essential to design efficient conservation and management strategies^1^. However, detecting subdivision and delineating units is often challenging in marine species due the lack of physical barriers to movement across wide geographic areas^2,3^ and the high dispersal potential of many species, as well as to the large sizes of marine populations, which leads to slow accumulation of genetic differences among units^3^. Pelagic species such as tunas combine life history characteristics that are likely to promote high connectivity. Most studies of tunas and other large pelagics have often shown weak or lack of structure across large geographic areas in some cases spanning entire oceanic basins^4–6^ highlighting the importance of studying connectivity at the scale of a species range to identify units when they exist.

Blackfin Tuna (*Thunnus atlanticus,* Lesson, 1830) is a small tuna found in tropical and subtropical waters of the Western Atlantic Ocean, ranging from the mid-Atlantic region of the United States east coast to northern Brazil^7,8^. Blackfin tuna are exploited commercially using purse seines, and various hook and line techniques (e.g., handline, vertical long lines, jigging, or trolling) in Brazil and in several parts of the Caribbean Sea, including Cuba, the Dominican Republic, the Lesser Antilles, and Venezuela^9–12^. The species is not commercially fished in the U.S. However, yearly recreational landings have increased in the past few decades from an average of 87,295 specimens for the period 1981–1985 to 294,256 specimens for the period 2015–2019 (Personal Communication of the National Marine Fisheries Service, Fisheries Statistics Division).

Blackfin tuna have been reported at depths reaching 700 m but are typically found within 50 m of the surface^13^. Factors affecting their distribution include water temperature and clarity, steepness of the continental shelf slope, and plankton concentrations, which have been related to upwelling and current rips, and runoff from land^14^. Spawning occurs offshore over epipelagic waters, from late spring to fall with a peak in early summer^15–17^. Blackfin tuna are commonly encountered closer to the shore outside of the spawning period, presumably feeding^18^.

Despite the commercial and recreational exploitation of blackfin tuna stocks, information on population biology and stock structure is still very limited and insufficient to develop reliable assessment and management of this species. Blackfin tunas, like other tunas, have a strong potential for dispersal at all life stages. They can disperse by pelagic passive transport of eggs and larvae after spawning^19^ and are also capable of long-distance movement at the juvenile and adult stages as reported for other tunas (e.g., transoceanic migrations of the closely related bluefin tuna^20^). Interestingly, conventional tagging studies in the areas of St Vincent and the Grenadines and Bermuda islands revealed that tagged fish were recaptured in close proximity of the tagging location^21,22^ even when recaptures occurred after multiple years (up to more than 3 years in the study of Singh Renton and Renton^22^), but individuals tagged off Bermuda were recaptured during the summer months only, suggesting the occurrence of migrations to southern portions of the range during the colder months^21^. Hypotheses proposed by Lukhurst et al.^21^ to explain these results were that (i) blackfin tuna may observe seasonal migrations from feeding areas to spawning areas and would show fidelity to specific feeding and spawning grounds, respectively, and (ii) some populations of blackfin tuna may tend to be more sedentary than others, as observed in other tunas^23^. In this context, the analysis of genetic stock structure in combination with approaches to study movement such as physical tagging and otolith microchemistry, is essential to reveal occurrences of differentiated genetics stocks and their habitat use, and to avoid overexploitation and depletion of some of them^1,24^.

To date, information on the population genetic structure and connectivity of blackfin tuna is limited to one study by Saxton^25^ who surveyed mitochondrial DNA and 6 heterologous microsatellites in the Gulf of Mexico and northwest Atlantic regions. The study revealed significant albeit low divergence between the two populations. The present study employs 13 of the microsatellite markers developed by Antoni et al.^26^ to investigate the genetic variation in blackfin tuna across 9 localities spanning most of the species range. The dataset was used to assess whether blackfin tuna are subdivided within their range and to test whether genetic heterogeneity reflected discrete units potentially separated by barriers or a continuous model driven by isolation by distance. The occurrence of cryptic structure was also assessed by testing temporal stability of spatial patterns and clustering approaches.

## Methods

### Tissue sampling

**Figure 1:**
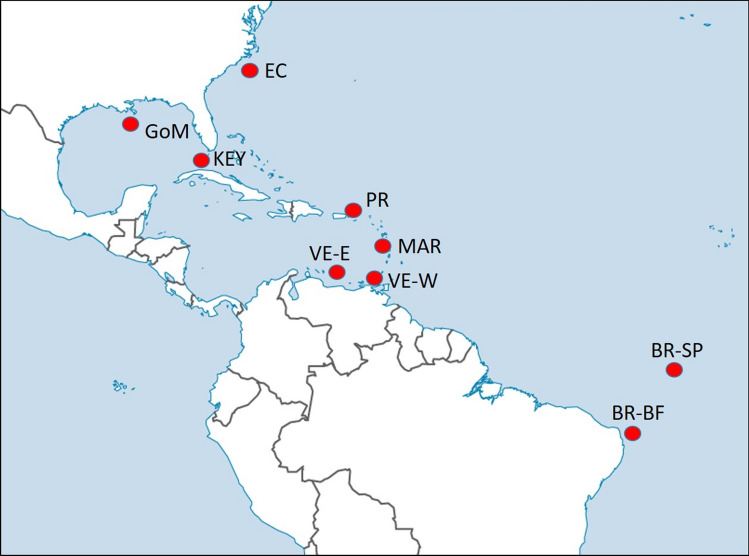
Sample localities for blackfin tuna (*Thunnus atlanticus*) in the western Atlantic Ocean. U.S. East coast Carolinas (EC), Florida Keys (Key), Gulf of Mexico Louisiana (GoM), Puerto Rico (PR), La Martinique Island (MAR), Venezuela (East sampling locality VE-E, West sampling locality VE-W), Brazil (Baía Formosa BR-BF, St Peter and St Paul BR-SP).

Tissue samples were obtained from a total of 470 blackfin tuna from nine offshore localities throughout the western Atlantic Ocean (Fig. 1, Table 1), including the US East Coast offshore North and South Carolina, the Florida Keys, the northern Gulf of Mexico offshore Louisiana, Puerto Rico offshore Fajardo, La Martinique Island, two collection areas in Venezuela, and two collection areas in Northeast Brazil (Baía Formosa in the Rio Grande do Norte State, and St Peter and St Paul archipelago).

**Table 1:** Sample sizes per year in nine localities surveyed for blackfin tuna (*Thunnus atlanticus*) in the western Atlantic Ocean.

	2012	2013	2014	2015
EC	10	77	–	–
Key	–	20	–	62
GoM	–	16	65	–
PR	–	37	20	–
MAR	–	61	–	–
VE-E	–	9	–	–
VE-W	–	50	–	–
BR-BF	–	20	–	–
BR-SP	–	23	–	–

U.S. East coast Carolinas (EC), Florida Keys (Key), Gulf of Mexico Louisiana (GoM), Puerto Rico (PR), La Martinique Island (MAR), Venezuela (East sampling locality VE-E, West sampling locality VE-W), Brazil (Baía Formosa BR-BF, St Peter and St Paul BR-SP).

Tissue samples were collected postmortem from fish carcasses during fisheries-dependent sampling. Tissue collections in La Martinique was conducted by IFREMER scientists during sampling activities part of the MAGDELESA project and authorized in the context of that project. The data used in this research, from Brazil, were obtained with the approval of the Instituto Chico Mendes de Conservação da Biodiversidade (ICMBio) of the Brazilian Ministry of the Environment (permit no. 53702) to Dr. Fabio Hazin. Tissue sample collections in US and Venezuelan waters were conducted on carcasses of fish of legal size caught during commercial fishing or recreational tournaments.

Sampling targeted adult specimens of reproductive size (over 50 cm Fork Length) that were captured by hook and line. Samples were obtained in two different years for four of the localities. Capture coordinates were not known for most samples, which were obtained from recreational or charter boats at landing. Capture was assumed to have occurred within a 100 miles radius of the landing port in those cases. The sampling was not stratified. Collection information specific to each individual sample is available along with genetic data in the University of Southern Mississippi public repository Aquila (https://aquila.usm.edu/datasets/6/).

Tissue samples, primarily fin clips, were stored in 95% ethanol or in DMSO salt-saturated fixative (0.5M EDTA, 20% dimethyl sulfoxide, NaCl, ddH_2_O) and returned to USM for analysis.

### Microsatellite genotyping

All samples were assayed at 13 of the microsatellite loci (BT4, BT11, BT18, BT20, BT22, BT27, BT29, BT31, BT68, BT81, BT83, BT88, and BT95) developed by Antoni et al.^26^. Seven loci initially developed by Antoni et al.^26^ were not used in this study because initial testing of these markers conducted during the early phases of the present study revealed high frequencies of scoring artifacts affecting genotyping. PCR primers used to amplify each microsatellite were combined in five multiplex panels for amplification and electrophoresis. PCRs were conducted in 6.48 or 6.92 mL reactions containing 7–13 ng of genomic DNA, 2.2 pmol of each primers, 8.4 nmol of MgCl_2_, 1.1 nmol of dNTPs, 0.28 U of GoTaq Flexi DNA Polymerase (Promega), and 1X of reaction buffer. The amplification cycle consisted in an initial denaturation at 95 °C for 5 min, 35 cycles at 95 °C for 30 s, annealing temperature for 30 s, 72 °C for 45 s, and a final extension at 72 °C for 10 min. The composition of each multiplex panel and annealing temperatures used during amplification are available on the Aquila public repository (https://aquila.usm.edu/datasets/6/). Microsatellite amplification products were electrophoresed using an ABI 377 automated sequencer (Applied Biosystems Inc., Foster City, CA), following the manufacturer’s instructions. Resulting chromatograms were analyzed in GENESCAN v. 3.1.2 (Applied Biosystems) and alleles were scored using GENOTYPER v. 2.5 (Applied Biosystems).

### Statistical analysis

Summary statistics for microsatellite data, including number of alleles (*A*), allelic richness (*A*_*R*_), expected heterozygosity (*H*_*E*_) (unbiased gene diversity), and the inbreeding coefficient *F*_*IS*_ (measured as Weir and Cockerham^27^ *f*) were generated for each sample locality and locus, using FSTAT v. 2.9.3.2 (available at http://www2.unil.ch/popgen/softwares/fstat.htm)^28^.

Tests of conformance of genotype frequencies to Hardy–Weinberg equilibrium (HWE) expectations were carried out for each microsatellite, using an exact probability test as implemented in GENEPOP v. 4.3^29^. The exact probability was estimated using a Markov Chain approach^30^ that employed 10,000 dememorizations, 500 batches and 5,000 iterations per batch. Occurrences of genotyping errors due to null-alleles, large allele dropout, or stuttering were evaluated for each microsatellite in each sample locality using MICROCHECKER v. 2.2.1^31^.

Analysis of molecular variance^32^ accounting for variation among localities and among years within locality was conducted in ARLEQUIN v. 3.5^33^. Samples were aggregated by sampling year within locality for this analysis. Significance of molecular variance components (among localities and between year-samples within locality) were assessed using 10,000 permutations of haplotypes.

The degree of divergence among samples (Fixation index F_*ST*_) was estimated as Weir and Cockerham’s^27^ *θ*, as implemented in FSTAT v. 2.9.3.2^28^. Homogeneity among samples (the probability that *θ* = 0) was tested using exact homogeneity tests computed in GENEPOP and using the same Monte Carlo Markov Chain as above. The Benjamini–Hochberg^34^ correction was applied for all multiple tests performed simultaneously.

Occurrence of cryptic structure within the dataset was further explored using Bayesian clustering implemented in STRUCTURE v. 2.3.4 (available at http://pritch.bsd.uchicago.edu/structure.html)^35,36^. Multiple Monte Carlo searches were run varying the number of putative clusters (*K*) from 2 to 9 in order to identify the most likely value for *K*. The correlated allele frequency setting was used in computations along with information on collection location used as prior to assist during clustering. The latter option improves recovery of structure within the data when genetic divergence among populations is weak^37^. Monte Carlo searches were run for 1.1 10^8^ steps with the first 10^7^ steps discarded as burnin.

Cryptic structure was also evaluated using discriminant analysis of principal components^38^ in ADEGENET v. 2.1.3^38^. The number of clusters (K) was determined by applying the find.cluster procedure with increasing values of K and minimizing the obtained Bayesian Information Criterion and the number of Principal Components retained for the analysis was determined using cross validation.

Spatial structure was examined using spatial analysis of molecular variance (SAMOVA)^39^. SAMOVA employs a simulated annealing algorithm to optimize allocation of *N* geographic populations into *K* groups (2 ≤ *K* < *N*) in order to maximize the proportion of total genetic variance due to genetic variation among the inferred groups. Computations were conducted in SAMOVA v. 2.0 (available at http://cmpg.unibe.ch/software/samova2/). One hundred simulated annealing processes were used to determine optimal allocation of the nine geographic samples into two, three, four, five, six, seven, or eight groups.

A Mantel test implemented in GENALEX v. 6.5^40^ was used to examine whether there was a significant correlation between genetic divergence and geographic distance. Isolation by distance was tested between geographic samples and using individual models. Genetic distance was estimated as *F*_*ST*_/(1-*F*_*ST*_) for Mantel tests implemented on samples grouped by localities and using the distance of Smouse and Peakall^41^ for individual models. Geographic distance was log transformed as recommended by Rousset^42^ to account for dispersal in a two dimensional habitat. Significance of the correlation was tested using 10,000 permutations of the distance matrices.

## Results

### Genetic diversity

Summary statistics for the 13 microsatellite loci in each sample locality are given in Supplemental file S1. The number of alleles per locus ranged from 11 to 58 and average expected heterozygosity across localities ranged from 0.582 to 0.951. Allelic richness and gene diversity did not differ significantly among localities (allelic richness: Χ^2^ = 3.47, df = 8, P = 0.902; gene diversity: Χ^2^ = 6.46, df = 8, P = 0.596). Departures from HWE were significant in15 out of 195 tests before correction for multiple tests controlling for a 5% false discovery rate but only one test remained significant after correction (KEYS at locus *BT*68, Supplemental file S1). Analyses of scoring artifacts in MICROCHECKER indicated nine possible occurrences of null alleles *(BT*4 in the GoM, BR-SP and KEY localities, *BT*18 in VE-W, *BT*20 in MAR, *BT*22 in VE-W, *BT*27 in NC, *BT*68 in KEYS and *BT*81 in MAR). None of the possible null allele cases was associated with significant departure from HWE. Based on these results, all the loci were kept for further analysis.

### Variation within and among localities

Analysis of molecular variance showed no significant variance among localities (F_CT_ = − 0.0017, P = 0.903) or among years within locality (F_SC_ = 0.0019, P = 0.067). Pairwise exact tests comparing samples collected in different years within the same locality also were non-significant (data not shown). Samples were pooled across sampling years for further analyses considering the non-significant variance between years and the relatively short interval between temporal samples (within two year, which is expected to be less than the generation time in the species considering that the reported size at first maturity^43^ is not attained until fish reach two years of age^10^). Exact tests of population differentiation (temporal samples pooled) across loci indicated significant heterogeneity among geographic locations (Χ^2^ = 39.2, df = 26, P = 0.046). The corresponding overall estimate of F_ST_ was very low (0.0004). Pairwise F_ST_ estimates averaged -0.0015 and were all less than 0.0039 (Table 2). Pairwise exact tests between geographic locality samples revealed 8 significant comparisons before correction for multiple tests. While none of the pairwise comparisons remained significant after FDR correction, four of the eight tests comparing the Brazil Baía Formosa population to others were significant before correction, and another two tests yielded P values lower than 0.078. The remaining two tests involving Baía Formosa compared this location to the samples from the West Venezuela and St Peter and St Paul archipelago and yielded probability values above 0.16 (Table 2).

**Table 2:** Pairwise F_ST_ estimates between blackfin tuna (*Thunnus atlanticus*) from 9 geographic populations (below diagonal) and probability that F_ST_ values differed significantly from zero (above diagonal).

	EC	Key	GoM	PR	MAR	VE-E	VE-W	BR-BF	BR-SP
EC	–	0.7679	0.6347	0.4110	0.1111	0.6296	0.5071	0.0653	0.3788
Key	− 0.0008	–	0.6830	0.4740	**0.0244**	0.4785	0.7555	**0.0345**	0.7233
GoM	− 0.0004	− 0.0004	–	0.1980	0.0548	0.3569	0.1602	0.0781	0.7971
PR	0.0008	0.0010	0.0006	–	**0.0234**	**0.0189**	0.7730	**0.0428**	0.2428
MAR	0.0015	0.0018	0.0017	0.0012	–	**0.0365**	0.5264	**0.0179**	0.1203
VE-E	− 0.0006	− 0.0005	− 0.0002	0.0025	0.0017	–	0.5114	**0.0354**	0.4970
VE-W	− 0.0004	− 0.0042	0.0012	− 0.0038	− 0.0050	− 0.0028	–	0.1627	0.1913
BR-BF	0.0022	0.0037	0.0027	0.0039	0.0006	0.0029	− 0.0053	–	0.3326
BR-SP	− 0.0001	− 0.0018	− 0.0021	0.0003	0.0009	− 0.0019	− 0.0027	− 0.0035	–

Bold font highlights significant probability values (*P* < 0.05) before correction for multiple tests. No p-value was significant after correction.

### Clustering analyses

The highest probability of the data was obtained for K = 2 during Bayesian clustering runs varying the value of K in STRUCTURE. All individuals had mixed ancestry close to 50% in the two clusters indicating lack of structure (Supplemental file S2).

**Figure 2:**
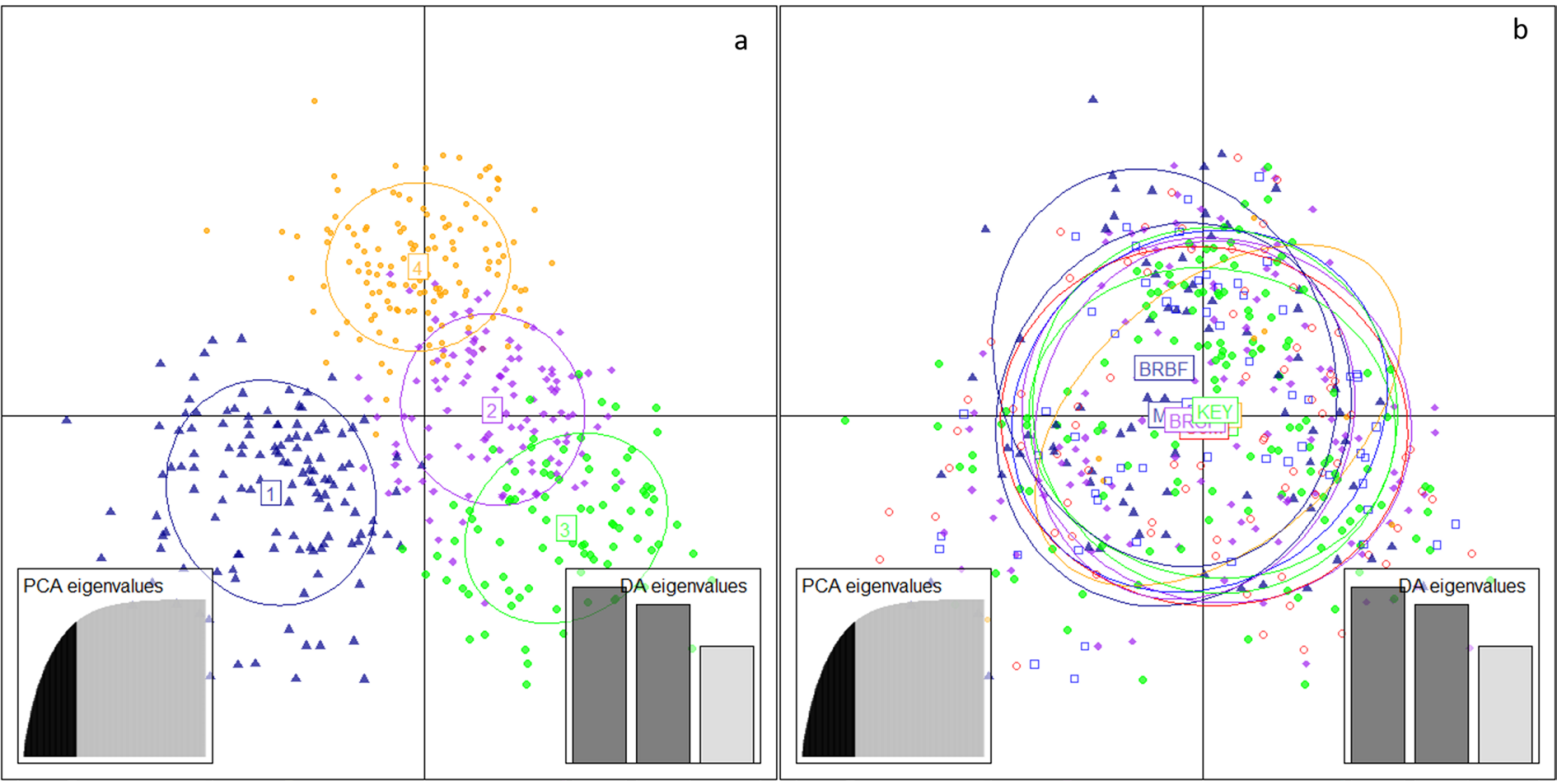
Discriminant analysis of principal components of the genotypes of 470 blackfin tunas assayed at 13 microsatellite markers. (**a**) Scatterplot representing the 4 inferred clusters on the first two components (**b**) representation of the same individuals grouped by locality samples on the same components showing overlapping confidence intervals.

One hundred principal components were retained for DAPC following cross validation, and the find.clusters procedure yielded 4 clusters (Fig. 2a). The four clusters were well separated by the first two discriminant components, but showed similar frequencies in all locality and temporal samples which were all aggregated at the centroid (Fig. 2b).

### Spatial analyses

The highest variance among groups was obtained with K = 2 groups during spatial analysis of molecular variance (F_CT_ = 0.009). The optimized partition of geographic samples separated the Brazil sample from Baía Formosa (BR-BF) in one group and all the rest of the samples in the second group. The significance of the among-group component of molecular variance could not be assessed because there were only nine different configurations of two groups comparable to the optimal configuration during permutations (comparable configurations had to feature one locality in one group and the other 8 localities in a second group)^44^, such that the lowest possible P-value was *P* = 0.114 (which was the observed P-value).

**Figure 3:**
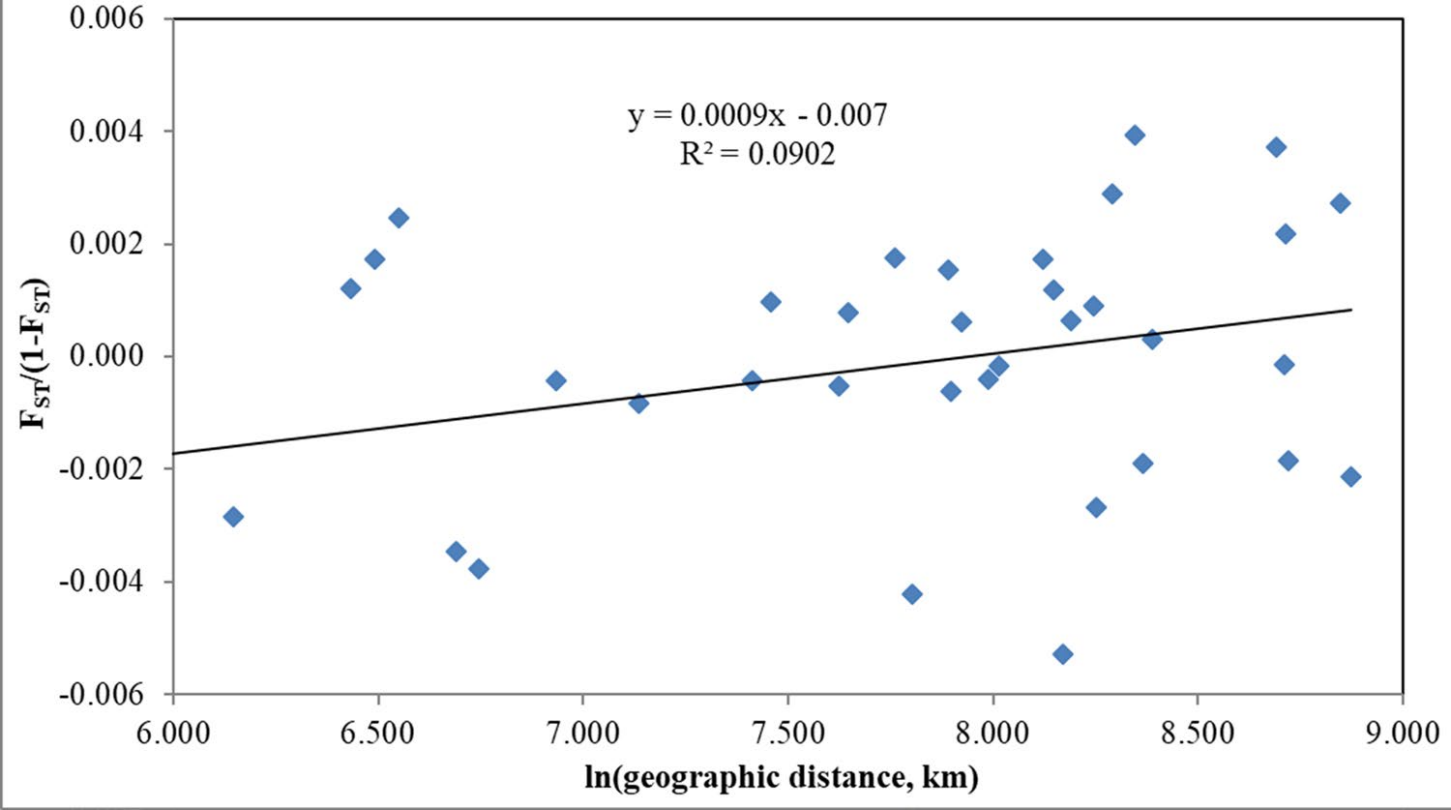
Plot of F_ST_/(1-F_ST_) as a function of ln geographic distance between localities.

The slope of the isolation by distance model was moderate but positive and the Mantel test indicated a significant association between genetic and geographic distances (*r* = 0.3, *P* = 0.040 for the population model, Fig. 3; *r* = 0.075, *P* = 0.002 for the individual model, data not shown). When the Baía Formosa sample was removed, the slopes of the population and individual models dropped to *r* = 0.103 (*P* = 0.404) and *r* = 0.040 (*P* = 0.039), respectively.

## Discussion

This manuscript reports the first survey of blackfin tuna population genetic structure across the species range. Considering their pelagic lifestyle and overall high potential for dispersal at all life stages, blackfin tuna were predicted to display high levels of connectivity across large distances. This hypothesis was supported by the observation of very weak levels of spatial divergence including an overall F_ST_ of 0.0004 and nonsignificant spatial components of molecular variance during hierarchical analysis of molecular variance.

A weak but significant positive correlation between genetic and geographic distances was found during Mantel tests. The divergence of the Baía Formosa from the rest of the range was driving part of the correlation, as the estimate of the isolation by distance slope was reduced when the Baía Formosa sample was removed from the analysis, but the slope of the individual model was still significant suggesting that blackfin tuna do not form a panmictic population across the northern part of the range but are structured, in part, according to geographic distance. The latter finding is consistent with some of the mark-recapture studies that indicated site fidelity across time^21,22^. Blackfin tuna are expected to reflect genetic variation patterns resulting in part from population expansion following the last glacial epoch, as discussed for other Atlantic pelagic species^45,46^. Their populations are likely large considering the high diversity recorded in this study with up to 58 alleles detected at microsatellite loci. The long time needed for genetic differences to accumulate under the effect of genetic drift in large populations combined with episodic gene flow events may further contribute to homogenizing populations that are otherwise isolated^47^, thereby maintaining apparent homogeneity as discussed for other species in the region^48,49^. In such a scenario, isolation by distance is expected to develop first between proximal localities^50^. Our sampling attempted to span the species range but had few population pairs separated by short or intermediate distances. Refining the spatial autocorrelation pattern would be of interest and will require sampling a larger number of localities, including some separated by shorter distances, to more effectively describe the isolation by distance model.

None of the pairwise exact tests were significant after correction for multiple tests but four of the eight tests comparing the Brazil Baía Formosa population to others were significant before correction and another two tests were close to significance (P < 0.078). Only tests comparing Baía Formosa to two of its closest geographic neighbor populations (St Peter and St Paul and West Venezuela) were insignificant. The divergence of the Baía Formosa geographic population was also suggested by spatial analysis of molecular variance, during which the most supported configuration separated Baía Formosa in one group from all the rest of the samples in a second group. The sample size of Baía Formosa was small (only 20 individuals); thus, the divergent status of this population will need to be confirmed with additional sampling, but collectively, the above results suggest that this population shows some degree of demographic independence from the other parts of the sampled range. Populations off the coast of Brazil, including the Baía Formosa area, are separated from the Caribbean region and the rest of the species range by the outflows of the Amazon River in northern Brazil and of the Orinoco River in eastern Venezuela. These barriers have been shown to effectively restrict gene flow for several reef fishes that disperse pelagic larvae^51,52^. If, as suggested by some of the results of tagging studies^21,22^, blackfin tuna exhibits site fidelity as adults and gene flow is mediated by larval dispersal, the Amazon and Orinoco barriers could also prevent connectivity between populations of this species located in Brazil and Caribbean ones. Another factor potentially acting to promote reproductive isolation between Brazilian and northern populations is the shift of the reproductive season which is reported to occur between November and March in the Southern Hemisphere^53^ but peaks between May and July in Northern Hemisphere stocks^12^. Accordingly, populations from the Northern and Southern Hemisphere may become naturally demographically independent due to a decoupling of spawning seasons where larvae spawned in the Southern Hemisphere in December do not find optimal conditions if they are transferred to northern regions. Similarly, ‘northern’ larvae spawned in June or July would find winter, potentially unfavorable, conditions if they were transferred to Brazil, although such a transfer seems unlikely considering the direction of the main surface current in the area (the North Brazil Current runs southeast to northwest, https://oceancurrents.rsmas.miami.edu/atlantic/north-brazil.html). In addition, adults migrating from one of the two stocks to the other may show limited reproductive activity post migration due to the shifting of reproductive periods. Additional sampling in South America and at the transition between Brazil and the Caribbean Sea is therefore warranted to confirm the demographic independence of the two regional stocks, describe stock boundaries, and identify factors restricting gene flow between the two regions. The St Peter and St Paul archipelago population did not diverge significantly from either the northern localities or the Baía Formosa population sample. This location is further north from Baía Formosa and may have received migrants from both ‘Northern Hemisphere’ and Brazilian populations, which would contribute to maintaining the St Peter and St Paul population similar to both groups. Considering the small sample size in the St Peter and St Paul area (23 individuals) and the very low divergence between the ‘Northern Hemisphere’ and Brazilian stocks, the divergence of St Peter and St Paul from either of the two other stocks may be too small to be detected with this dataset. The archipelago is very close to the equator line and, while spawning in this area seems to be more active in the first semester as in Brazil, actively spawning individuals are found almost all year round^43^, suggesting that adult migrants of both northern and southern origin would find conditions conducive to spawning and that their progeny would also find favorable conditions for development leading to successful incorporation of migrant genotypes in the local gene pool. Finally, pairwise comparisons also revealed marginal differences between La Martinique Island and the Gulf of Mexico, Puerto Rico the Keys and East Venezuela (and Baía Formosa). La Martinique did not differ significantly from the larger (West) Venezuela sample, St Peter & St Paul or the US East coast; thus, these small differences in allele frequencies are more difficult to interpret but could possibly signal some degree of isolation of blackfin tuna in the lesser Antilles region.

Population structuring related to natural selection has been detected in a number of marine species even where neutral variation was very low^54,55^, a scenario that may apply to blackfin tuna as well. Variation due to selection and adaptation may not be strictly geographic but could also be cryptic related to parameters of the landscape or habitat characteristics such as temperature and habitat use. This study only employed 13 markers which is insufficient to provide information on the effects of selection. Examination of regions of the genome influenced by selection through the implementation of high-density genome scans is therefore warranted for future studies. We note that the among-years component of molecular variance was close to significant and larger than the spatial (among localities) component which suggests that some form of cryptic structure might exist. The occurrence of cryptic structure was examined using model-based Bayesian clustering in STRUCTURE and discriminant analysis of principal components. Model-based Bayesian clustering failed to identify subunits within the dataset. While the levels of divergence between such units, if they exist, are unknown, the divergence between geographic samples was very weak (below 0.005), an order of magnitude below those that can be detected by STRUCTURE^56^. Discriminant analysis of principal components supported the occurrence of four clusters. While these clusters might indicate the occurrence of some form of cryptic structure, the distribution of cluster membership was uncorrelated to the spatial origin of the samples in this study and this analysis may reflect artifacts considering the weak divergence across the sampling surface. However, cryptic structure within the dataset is possible and could be related to groups featuring different habitat use and movement patterns. This study sampled adults of reproductive size but could not confirm whether specimens were collected on or close to their spawning grounds. Sampling nursery sites in future studies would be useful to better assess the breeding structure of blackfin tuna as was successfully done in other tunas^57^.

The lack of significant subdivision in US waters in this study contrasts with the findings of Saxton^25^ who detected statistically significant, yet low, divergence between the Gulf of Mexico and the NW Atlantic populations. Saxton^25^ used only 6 heterologous microsatellites and one mitochondrial gene and samples were taken in 1994–1995 for the East US coast and 2001–2007 for the Gulf of Mexico such that spatial variations between the two regions were potentially confounded by temporal variations. This study addressed these limitations by employing a larger panel of homologous loci and more comprehensively sampling the species range. Fine-scale structuring within US waters as suggested by Saxton^25^ may occur but is likely very weak considering the results of the present study.

In conclusion, this study indicates the occurrence of weak spatial structuring of blackfin tuna populations, which warrants further investigation to determine if management of the resource needs to account for multiple stocks. Isolation by distance throughout the range was suggested by the outcome of the Mantel tests and will need further description with finer geographic sampling to determine the spatial scale of dispersal and the distance at which stocks are expected to be demographically independent. While the results suggest divergence of the Brazilian and Northern Hemisphere populations, further research is needed to confirm the occurrence of distinct stocks, their geographic delineation, and their relationships, in particular their degree and pattern of connectivity and the mechanisms involved in isolation. The analysis of high-density genome scans in future studies may prove useful to improve inference power for the detection of differentiated stocks and assess variation under selection.

## Supplementary Information

Supplementary Information 1.

Supplementary Information 2.

## Data Availability

Data are available on the Aquila repository of the University of southern Mississippi available at https://aquila.usm.edu/datasets/6/.
