# Supplementary material for: Population structure of blackfin tuna (Thunnus atlanticus) in the western Atlantic Ocean inferred from microsatellite loci
Source: Sci Rep. 2022 Jun 14;12:9830. doi: 10.1038/s41598-022-13857-z (PMC9198023; doi:10.1038/s41598-022-13857-z)
Supplement: Supplementary file 1 — Supplementary Information 1. [file 41598_2022_13857_MOESM1_ESM.docx]

**Supporting information**

**Supplemental file S1**. Summary statistics for 13 nuclear-encoded microsatellites in blackfin tuna, [*Thunnus*](http://www.fishbase.org/summary/speciessummary.php?id=188) *atlanticus*, sampled from the US east coast (EC), the Florida Keys (KEY), the northcentral Gulf of Mexico (GoM), Puerto Rico (PR), La Martinique Island (MAR), two localities in Venezuela (VE-E and VE-W), and two localities in northern Brazil (BR-BF and BR-SP). *N*, sample size, *#A*, number of alleles, *A_R_*, allelic richness*, H_E_*, gene diversity (expected heterozygosity), *P_HW_*, probability of conforming to expected Hardy–Weinberg genotypic proportions, *F_IS_*, inbreeding coefficient measured as Weir and Cockerham’s (1984) *f*.

| **Locus** | **EC** | **KEY** | **GoM** | **PR** | **MAR** | **VE-E** | **VE-W** | **BR-BF** | **BR-SP** |
| --- | --- | --- | --- | --- | --- | --- | --- | --- | --- |
| *BT*04 |  |  |  |  |  |  |  |  |  |
| *N* | 87 | 81 | 79 | 52 | 58 25 19 7 21 19 12 12 24 58 | 9 | 50 | 20 | 23 |
| *#A* | 25 | 24 | 21 | 19 | 19 | 7 | 19 | 12 | 12 |
| *A_R_* | 9.572 | 9.123 | 9.286 | 8.897 | 9.390 | 7.000 | 9.030 | 8.189 | 8.055 |
| *H_E_* | 0.888 | 0.881 | 0.887 | 0.868 | 0.893 | 0.792 | 0.877 | 0.847 | 0.856 |
| *P_HW_* | 0.2413 | 0.0224 | 0.0182 | 0.7809 | 0.0373 | 0.5475 | 0.313 | 0.15 | 0.005 |
| *F_IS_* | 0.068 | 0.117 | 0.116 | 0.070 | 0.054 | 0.018 | -0.050 | 0.115 | 0.289 |
| *BT*11 |  |  |  |  |  |  |  |  |  |
| *N* | 86 | 81 | 81 | 57 | 61 | 9 | 50 | 20 | 21 |
| *#A* | 33 | 31 | 31 | 27 | 25 | 14 | 26 | 18 | 19 |
| *A_R_* | 12.846 | 12.404 | 12.248 | 12.599 | 11.821 | 14.000 | 12.021 | 11.964 | 12.658 |
| *H_E_* | 0.956 | 0.951 | 0.949 | 0.954 | 0.945 | 0.972 | 0.948 | 0.949 | 0.958 |
| *P_HW_* | 0.3876 | 0.1532 | 0.048 | 0.2157 | 0.502 | 0.3105 | 0.1216 | 0.1185 | 0.8144 |
| *F_IS_* | 0.027 | 0.014 | 0.037 | 0.007 | 0.028 | 0.086 | 0.050 | -0.001 | 0.006 |
| *BT*18 |  |  |  |  |  |  |  |  |  |
| *N* | 87 | 81 | 81 | 57 | 59 | 9 | 50 | 20 | 23 |
| *#A* | 22 | 22 | 20 | 19 | 18 | 8 | 17 | 12 | 17 |
| *A_R_* | 9.961 | 10.830 | 10.270 | 9.738 | 9.959 | 8.000 | 10.642 | 9.122 | 11.165 |
| *H_E_* | 0.913 | 0.931 | 0.919 | 0.901 | 0.917 | 0.889 | 0.932 | 0.903 | 0.939 |
| *P_HW_* | 0.1474 | 0.2738 | 0.9811 | 0.6183 | 0.1905 | 0.5345 | 0.1752 | 0.2105 | 0.6642 |
| *F_IS_* | 0.056 | 0.072 | -0.007 | -0.013 | 0.002 | 0.000 | 0.099 | -0.052 | 0.074 |
| *BT*20 |  |  |  |  |  |  |  |  |  |
| *N* | 87 | 81 | 80 | 54 | 61 | 9 | 50 | 20 | 22 |
| *#A* | 21 | 23 | 24 | 19 | 26 | 11 | 21 | 14 | 16 |
| *A_R_* | 10.512 | 11.246 | 10.931 | 9.561 | 11.554 | 11.000 | 10.942 | 9.884 | 10.771 |
| *H_E_* | 0.923 | 0.933 | 0.929 | 0.907 | 0.938 | 0.931 | 0.933 | 0.917 | 0.933 |
| *P_HW_* | 0.5391 | 0.38 | 0.219 | 0.395 | 0.0764 | 0.6435 | 0.0868 | 0.5936 | 0.2499 |
| *F_IS_* | 0.041 | -0.019 | -0.022 | -0.000 | 0.091 | -0.075 | 0.035 | 0.073 | 0.026 |
| *BT*22 |  |  |  |  |  |  |  |  |  |
| *N* | 87 | 81 | 81 | 56 | 61 | 9 | 50 | 20 | 23 |
| *#A* | 31 | 29 | 30 | 28 | 25 | 10 | 22 | 13 | 16 |
| *A_R_* | 10.061 | 10.108 | 9.443 | 10.677 | 10.016 | 10.000 | 9.926 | 8.842 | 9.846 |
| *H_E_* | 0.904 | 0.905 | 0.892 | 0.920 | 0.909 | 0.938 | 0.907 | 0.882 | 0.903 |
| *P_HW_* | 0.1898 | 0.1975 | 0.0854 | 0.4277 | 0.0281 | 0.2303 | 0.0063 | 0.3191 | 0.3986 |
| *F_IS_* | 0.034 | -0.051 | -0.038 | -0.028 | 0.062 | 0.170 | 0.118 | -0.021 | -0.011 |
|  |  |  |  |  |  |  |  |  |  |
| *BT*27 |  |  |  |  |  |  |  |  |  |
| *N* | 84 | 81 | 80 | 55 | 59 | 9 | 50 | 14 | 19 |
| *#A* | 19 | 20 | 20 | 17 | 18 | 7 | 19 | 10 | 15 |
| *A_R_* | 9.425 | 9.164 | 9.353 | 8.583 | 9.281 | 7.000 | 9.678 | 8.971 | 10.672 |
| *H_E_* | 0.904 | 0.898 | 0.904 | 0.878 | 0.895 | 0.882 | 0.909 | 0.912 | 0.934 |
| *P_HW_* | 0.0046 | 0.2947 | 0.1494 | 0.2103 | 0.0945 | 0.9052 | 0.0912 | 0.7471 | 0.2648 |
| *F_IS_* | 0.118 | -0.031 | -0.023 | 0.026 | -0.003 | -0.134 | 0.054 | -0.096 | 0.099 |
| *BT*29 |  |  |  |  |  |  |  |  |  |
| *N* | 87 | 81 | 81 | 56 | 61 | 9 | 50 | 18 | 23 |
| *#A* | 25 | 24 | 26 | 24 | 22 | 11 | 23 | 17 | 19 |
| *A_R_* | 11.154 | 10.069 | 11.437 | 11.153 | 10.751 | 11.000 | 11.671 | 11.703 | 11.264 |
| *H_E_* | 0.930 | 0.909 | 0.937 | 0.927 | 0.920 | 0.951 | 0.941 | 0.948 | 0.929 |
| *P_HW_* | 0.7493 | 0.5308 | 0.6196 | 0.4997 | 0.6784 | 0.1689 | 0.5154 | 0.0049 | 0.0652 |
| *F_IS_* | 0.011 | -0.032 | -0.001 | -0.002 | 0.002 | 0.182 | -0.042 | 0.179 | 0.064 |
| *BT*31 |  |  |  |  |  |  |  |  |  |
| *N* | 86 | 81 | 79 | 55 | 60 | 9 | 50 | 16 | 23 |
| *#A* | 23 | 20 | 19 | 20 | 14 | 9 | 17 | 12 | 11 |
| *A_R_* | 9.423 | 9.598 | 9.431 | 9.913 | 8.045 | 9.000 | 9.433 | 9.243 | 8.217 |
| *H_E_* | 0.890 | 0.900 | 0.895 | 0.908 | 0.869 | 0.903 | 0.891 | 0.890 | 0.881 |
| *P_HW_* | 0.5241 | 0.9372 | 0.5047 | 0.559 | 0.3049 | 0.4919 | 0.6683 | 0.333 | 0.4195 |
| *F_IS_* | -0.032 | -0.043 | 0.038 | -0.021 | 0.041 | 0.015 | -0.011 | 0.087 | 0.063 |
| *BT*68 |  |  |  |  |  |  |  |  |  |
| *N* | 87 | 81 | 80 | 54 | 60 | 9 | 50 | 20 | 23 |
| *#A* | 10 | 10 | 10 | 10 | 10 | 7 | 10 | 10 | 9 |
| *A_R_* | 6.345 | 6.859 | 6.495 | 6.894 | 6.221 | 7.000 | 6.840 | 7.362 | 6.773 |
| *H_E_* | 0.827 | 0.843 | 0.822 | 0.843 | 0.806 | 0.868 | 0.841 | 0.851 | 0.832 |
| *P_HW_* | 0.315 | **0.0038** | 0.1342 | 0.1118 | 0.4242 | 0.79 | 0.227 | 0.2949 | 0.746 |
| *F_IS_* | -0.070 | 0.121 | 0.072 | -0.011 | 0.029 | 0.104 | -0.022 | -0.175 | -0.097 |
| *BT*81 |  |  |  |  |  |  |  |  |  |
| *N* | 87 | 81 | 81 | 55 | 60 | 9 | 50 | 20 | 23 |
| *#A* | 13 | 12 | 10 | 11 | 13 | 7 | 10 | 11 | 10 |
| *A_R_* | 6.859 | 6.647 | 6.423 | 6.498 | 7.221 | 7.000 | 6.878 | 8.123 | 7.138 |
| *H_E_* | 0.819 | 0.805 | 0.803 | 0.830 | 0.852 | 0.868 | 0.833 | 0.878 | 0.836 |
| *P_HW_* | 0.4099 | 0.3852 | 0.7458 | 0.0059 | 0.0202 | 0.4515 | 0.7771 | 0.5829 | 0.2937 |
| *F_IS_* | 0.032 | 0.019 | 0.001 | 0.036 | 0.159 | 0.104 | 0.040 | 0.145 | 0.012 |
| *BT*83 |  |  |  |  |  |  |  |  |  |
| *N* | 87 | 82 | 81 | 57 | 61 | 9 | 50 | 20 | 23 |
| *#A* | 8 | 8 | 9 | 8 | 8 | 4 | 8 | 8 | 7 |
| *A_R_* | 4.556 | 4.990 | 5.197 | 5.302 | 5.228 | 4.000 | 4.940 | 6.485 | 5.301 |
| *H_E_* | 0.532 | 0.582 | 0.620 | 0.618 | 0.658 | 0.611 | 0.547 | 0.780 | 0.609 |
| *P_HW_* | 0.6211 | 0.019 | 0.159 | 0.029 | 0.9924 | 0.804 | 0.2684 | 0.5905 | 0.3138 |
| *F_IS_* | -0.037 | 0.058 | 0.024 | 0.035 | -0.047 | 0.091 | 0.013 | 0.039 | 0.071 |
| *BT*88 |  |  |  |  |  |  |  |  |  |
| *N* | 87 | 81 | 81 | 56 | 61 | 9 | 50 | 17 | 23 |
| *#A* | 22 | 20 | 22 | 19 | 24 | 10 | 19 | 13 | 16 |
| *A_R_* | 10.573 | 10.605 | 10.407 | 10.277 | 10.810 | 10.000 | 10.596 | 9.835 | 10.770 |
| *H_E_* | 0.927 | 0.926 | 0.919 | 0.917 | 0.922 | 0.938 | 0.925 | 0.917 | 0.933 |
| *P_HW_* | 0.9877 | 0.6087 | 0.4588 | 0.3688 | 0.7496 | 0.6236 | 0.8507 | 0.6428 | 0.1264 |
| *F_IS_* | 0.008 | -0.027 | -0.035 | -0.012 | -0.067 | 0.052 | -0.017 | 0.038 | 0.114 |
| *BT*95 |  |  |  |  |  |  |  |  |  |
| *N* | 87 | 81 | 81 | 55 | 61 | 9 | 50 | 20 | 23 |
| *#A* | 17 | 15 | 15 | 12 | 17 | 10 | 13 | 10 | 10 |
| *A_R_* | 8.697 | 8.569 | 8.829 | 8.367 | 9.124 | 10.000 | 9.023 | 8.438 | 7.933 |
| *H_E_* | 0.883 | 0.877 | 0.891 | 0.887 | 0.903 | 0.938 | 0.904 | 0.899 | 0.871 |
| *P_HW_* | 0.2566 | 0.154 | 0.1056 | 0.0041 | 0.2253 | 0.2071 | 0.5259 | 0.119 | 0.5481 |
| *F_IS_* | 0.050 | 0.071 | 0.030 | 0.057 | 0.092 | 0.052 | 0.027 | 0.054 | 0.051 |

Bold font indicates a significant probability value after correction for multiple tests.
