# Supplementary material for: Population structure of blackfin tuna (Thunnus atlanticus) in the western Atlantic Ocean inferred from microsatellite loci
Source: Sci Rep. 2022 Jun 14;12:9830. doi: 10.1038/s41598-022-13857-z (PMC9198023; doi:10.1038/s41598-022-13857-z)

**Supporting information**

Supplemental file S2. Proportion of ancestry in two clusters obtained in Structure.


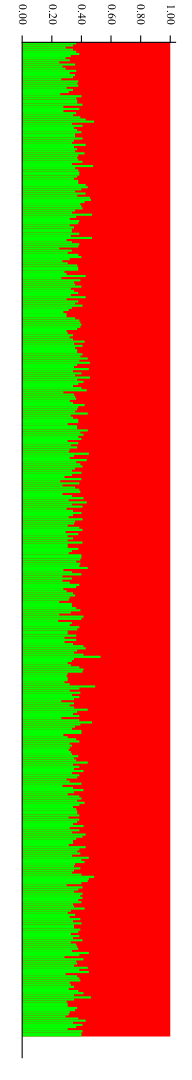

Supplement: Supplementary file 2 — Supplementary Information 2. [file 41598_2022_13857_MOESM2_ESM.docx]
